# Anatomic accuracy, physiologic characteristics, and fidelity of very low birth weight infant airway simulators

**DOI:** 10.1038/s41390-021-01823-w

**Published:** 2021-11-08

**Authors:** Patricia Lengua Hinojosa, Frank Eifinger, Michael Wagner, Jochen Herrmann, Monika Wolf, Chinedu Ulrich Ebenebe, Axel von der Wense, Philipp Jung, Aram Mai, Bettina Bohnhorst, Ann Carolin Longardt, Georg Hillebrand, Susanne Schmidtke, Florian Guthmann, Martina Aderhold, Ida Schwake, Maria Sprinz, Dominique Singer, Philipp Deindl

**Affiliations:** 1 Department of Neonatology and Pediatric Intensive Care Medicine, University Children’s Hospital, University Medical Center Hamburg-Eppendorf, Hamburg, Germany; 2 Department of Pediatric Critical Care Medicine and Neonatology, Faculty of Medicine and University Hospital Cologne, University of Cologne, Cologne, Germany; 3 Division of Neonatology, Pediatric Intensive Care and Neuropediatrics, Department of Pediatrics, Comprehensive Center for Pediatrics, Medical University of Vienna, Vienna, Austria; 4 Department of Diagnostic and Interventional Radiology and Nuclear Medicine, Section of Pediatric Radiology, University Medical Center Hamburg-Eppendorf, Hamburg, Germany; 5 Department of Neonatology, Children’s Hospital Hamburg-Altona, Hamburg, Germany; 6 University Children’s Hospital, University Hospital Schleswig-Holstein, Lübeck, Germany; 7 Department of Neonatology and Pediatric Intensive Care Medicine, Westcoast Hospital Heide, Heide, Germany; 8 Department of Pediatric Pulmonology, Allergology and Neonatology, Hannover Medical School, Hannover, Germany; 9 University Children’s Hospital I, Neonatology, University Hospital Schleswig-Holstein, Kiel, Germany; 10 Department of Neonatal Care, Hospital Itzehoe, Itzehoe, Germany; 11 Departement of Neonatal Care, Asklepios Hospital Barmbek & Nord, Hamburg, Germany; 12 Department of Neonatology, Children’s and Youth Hospital Auf der Bult, Hannover, Germany; 13 Department of Neonatal Care, Hospital Lüneburg, Lüneburg, Germany

## Abstract

**Background:**

Medical simulation training requires realistic simulators with high fidelity. This prospective multi-center study investigated anatomic precision, physiologic characteristics, and fidelity of four commercially available very low birth weight infant simulators.

**Methods:**

We measured airway angles and distances in the simulators Premature AirwayPaul (SIMCharacters), Premature Anne (Laerdal Medical), Premie HAL S2209 (Gaumard), and Preterm Baby (Lifecast Body Simulation) using computer tomography and compared these to human cadavers of premature stillbirths. The simulators’ physiologic characteristics were tested, and highly experienced experts rated their physical and functional fidelity.

**Results:**

The airway angles corresponded to those of the reference cadavers in three simulators. The nasal inlet to glottis distance and the mouth aperture to glottis distance were only accurate in one simulator. All simulators had airway resistances up to 20 times higher and compliances up to 19 times lower than published reference values. Fifty-six highly experienced experts gave three simulators (Premature AirwayPaul: 5.1 ± 1.0, Premature Anne 4.9 ± 1.1, Preterm Baby 5.0 ± 1.0) good overall ratings and one simulator (Premie HAL S2209: 2.8 ± 1.0) an unfavorable rating.

**Conclusion:**

The simulator physiology deviated significantly from preterm infants’ reference values concerning resistance and compliance, potentially promoting a wrong ventilation technique.

**Impact:**

- Very low birth weight infant simulators showed physiological properties far deviating from corresponding patient reference values.
- Only ventilation with very high peak pressure achieved tidal volumes in the simulators, as aimed at in very low birth weight infants, potentially promoting a wrong ventilation technique.
- Compared to very low birth weight infant cadavers, most tested simulators accurately reproduced the anatomic angular relationships, but their airway dimensions were relatively too large for the represented body.
- The more professional experience the experts had, the lower they rated the very low birth weight infant simulators.

## Introduction

### Background

Airway management of very low birth weight (VLBW) neonates is a crucial skill.^1^ The anatomical proportions in VLBW infants are narrow and require expertness and practice in all airway management techniques, including mask ventilation via a bag or t-device, intubation, laryngeal mask airway, and the less-invasive surfactant administration (LISA) method. LISA is used to treat respiratory distress syndrome in premature neonates by inserting a special catheter through the glottis under laryngoscopic visualization and applying surfactant during spontaneous breathing.^2,3^

In the past, physicians acquired airway management techniques through clinical practice. In the last years, primary invasive ventilation and endotracheal intubation have been increasingly replaced by non-invasive respiratory support and stabilization of VLBW infants.^4^ As a result, opportunities to practice complex airway management on real VLBW infants are becoming rarer, and the relevance of realistic high-fidelity simulators in medical education and clinical training raises.^5–8^ Simulators may be a helpful alternative to practice complex procedures, including intubation, without harming or endanger patients.^5,9,10^ A high simulator quality and fidelity (degree of exactness with which something is reproduced) are vital for effective medical education and clinical training in neonatology.^11–14^

Two different dimensions of simulator fidelity are described, (a) physical fidelity, and (b) functional fidelity.^15–17^ Physical fidelity reflects the quality of the simulator to imitate physical characteristics like haptic and optical impressions. Functional fidelity describes the simulator’s ability to demonstrate complex scenarios. According to Curtis et al.,^17^ high physical fidelity is crucial for developing psychomotor skills and functional fidelity likewise to build cognitive comprehension and sovereignty.

Several studies analyzed the anatomic accuracy of adult simulators, comparing them with humans.^18–20^ They showed that some simulators suffered from significant inaccuracies with incorrect airway dimensions, which may negatively affect training and cause over-confidence in users. Sawyer et al.^21^ analyzed the fidelity of eight neonatal airway simulators through expert review and found significant differences in expert ratings. Several commercially available high-fidelity airway simulators for VLBW infants under 1500 g body weight have been developed in the recent years.

### Aims

This prospective multi-center study aimed to investigate the anatomic precision, the physiologic airway characteristics, and the physical and functional fidelity of VLBW infant airway simulators.

## Materials and methods

We tested three aspects of simulator quality: (a) anatomic precision compared to human cadavers, (b) airway and lung physiology, and (c) fidelity according to experts in the following simulators: Premature Anne (Laerdal Medical, Stavanger, Norway), Premature AirwayPaul (SIMCharacters, Vienna, Austria), Premie HAL S2209 (Gaumard, Miami, Florida), and Preterm Baby (Lifecast Body Simulation, London, Great Britain). Detailed simulator characteristics are shown in Table 1.

**Table 1:** Very low birth weight infant airway simulator and corresponding reference cadaver characteristics.

Simulator model (manufacturer)	*n*	Gestational age (weeks)	Weight (g)	Weight percentile	Length (cm)	Length percentile	Head circumference (cm)	Head circumference percentile
Premature Anne (Laerdal Medical)	1	25	440	2	30	18	23	37
Premature AirwayPaul (SimCharacters)	1	27	1000	50	35	40	26	57
Preterm Baby (Lifecast Body Simulation)	1	28	980	39	36	42	24	14
Premie HAL S2209 (Gaumard)	1	30	1490	55	46	95	33	99
**Cadaver category**								
700–1000 g	3	25.8	916	63	37.3	79	25.8	67
1200–1400 g	2	27.2	1300	89	38.5	78	24.0	19
1400–1700g	2	29.2	1500	77	41.5	78	29.3	59

Weight, length, head circumference, and percentiles are reported as mean values for each reference cadaver category. Percentiles are calculated based on Voigt et al..^29^

### Anatomic precision of simulator airway dimensions

**Fig. 1:**
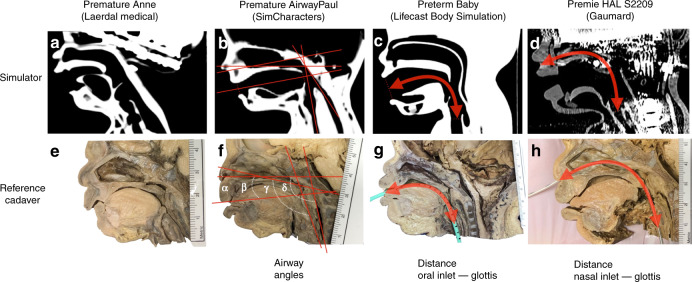
Anatomic precision of the tested simulator airways dimensions compared with human cadavers: **a**–**d** Computed tomography sagittal plane of the very low birth weight infant airway simulators. **e**–**h** Sagittal sections of anatomical specimens of premature stillborn infants with corresponding birth weight and gestational age. We measured the angles between the bony palate and (alpha) the nasal inlet, (beta) the mouth inlet, (gamma) the trachea, and (delta) the esophagus (**b**, **f**), the distance from the oral inlet to the glottis (**c**, **g**) for the oral tube position, and the distances from the nasal inlet to the glottis (**d**, **h**) for the nasal tube position. The baseline was placed as a tangent across the hard and soft palate. The line through the nasal inlet was placed centrally in the nasal ostium and then along the inferior conchae. The mouth opening was centered in the mouth inlet in the junction with the mouth outlet below the uvula. The lines through the trachea and esophagus were placed in their respective averaged long axes. In each case, a simulator and an anatomical specimen are shown as examples. In order to standardize the orientation in the figure, some images and the metric scale have been flipped horizontally.

We measured simulator airway dimensions by computer tomography (CT) and compared angles and distances between important anatomic airway structures with anatomical preparations of stillborn, premature infant cadavers of corresponding gestational age, and body measurements (Table 1). Comparison of the Premature Anne simulator (Laerdal), which represents a premature infant with a body weight of 440 g, was complicated because we had no suitable reference cadavers available. We, therefore, compared Premature Anne (Laerdal) with heavier reference cadavers (700–1000 g). The cadavers used were legal donations (ethics vote of the University of Cologne, No. 16-408) (Fig. 1). The simulators were scanned on a computer tomograph (iQon Spectral CT, Philips Healthcare, Release version 4.7.5), and multi-planar reconstructions in 1 mm slice thickness were performed. Distances and angles were measured on a sagittal midplane reconstruction in the soft tissue window depicting the nasopharyngeal inlet and the glottis region on one image. The level of the glottis was determined on transverse images. We measured the angles between the tangential of the bony palate and (alpha) the nasal inlet, (beta) the mouth inlet, (gamma) the trachea, and (delta) the esophagus in the sagittal plane and the distances between the nasal and oral inlet and the glottis (Fig. 1). We used three cadavers as reference for Premature AirwayPaul (SIMCharacters) and Premature Anne (Laerdal Medical) (birth weights: 0.75, 0.9, 1.1 kg, gestational age: 25 + 0, 26 + 0, 26 + 3 weeks), two cadavers as reference for Premie HAL S2209 (Gaumard) (birth weights: 1.4, 1.6 kg gestational age: 29 + 1, 29 + 2 weeks), and two cadavers as reference for Preterm Baby (Lifecast Body Simulation) (birth weights: 1.2 kg, 1.3 kg, gestational age: 26 + 6, 26 + 5 weeks). According to their respective percentiles, the reference cadavers had normal body weights, body lengths, and head circumferences, whereas Premature Anne (Laerdal Medical) was very light for the indicated gestational week, and Premie HAL (Gaumard) had a very large head circumference (see Table 1).

### Simulator airway and lung physiology

We measured the simulator’s airways and lungs’ physiological characteristics using the Leonie Plus neonatal ventilator (Heinen and Löwenstein, Bad Ems, Germany). The respective simulator airways were connected leak-free to the ventilator via a 2.5 mm tube (Vygon, Aachen, Germany) using a commercially available ventilation hose system (Fisher & Paykel, Schorndorf, Germany). We measured inspiratory tidal volume, minute volume, resistance, and compliance of the simulators at three different ventilator settings (low, moderate, high) using constant settings for the inspiratory time of 0.3 s, oxygen fraction of 0.21, frequency of 50/min, and a gas flow of 8 L/min: (a) peak inspiratory pressure (Pip): low: 15 cm H_2_O, positive end-expiratory pressure (PEEP) 5 cm H_2_O; (b) moderate: Pip 20 cm H_2_O, PEEP 6 cm H_2_O; (c) high: Pip 25 cm H_2_O, PEEP 7 cm H_2_O. We ventilated the simulators at moderate intensity using three different tube diameters (2.0, 2.5, and 3.0 mm) to measure the air leak. We performed each physiologic measurement three times.

### Expert ratings of simulator fidelity

We showed experienced neonatologists (experts) from hospitals with different care levels high-resolution pictures of the mouth, pharynx, epiglottis, and glottis of the simulators captured with a video laryngoscope (model C-MAC, Storz, Tuttlingen, Germany) in a randomized order (SFig. 1). After each set of photos, the experts were requested to state how they rated the fidelity of the skin, face, mouth, gingiva, tongue, glottis, vocal cords, airway, and anatomy of the simulator using a questionnaire. We then asked the experts to perform the following airway maneuvers: (a) bag-mask ventilation, (b) oral intubation, (c) nasal intubation, (d) less-invasive surfactant administration on each simulator in randomized order, and to rate the simulators’ functional fidelity. All aspects of simulator quality were scored on a scale of absolutely unrealistic (1), unrealistic (2), somewhat unrealistic (3), undecided (4), somewhat realistic (5), realistic (6), and absolutely realistic (7), corresponding to a score from 1 to 7. Besides, we used a questionnaire to elicit professional status, years of professional experience, and information on the number of airway management maneuvers performed in preterm infants to assess the experts’ expertise.

### Statistical analysis

Statistical analyses were performed using R 4.0.3 (2020-10-10) (R Core Team, Vienna, Austria). We calculated the minimum number of experts to be 43 based on the following assumptions: mean overall rating differs on average by 1, a standard deviation of 0.5, a power of 0.9, and a significance level of 0.05. We calculated means and standard deviations (SD) for the expert rating. We applied a linear regression model including professional experience, the number of participants per center, hospital care level, and simulator type as predictors of the overall expert rating. Continuous variables are expressed as means ± standard deviations (SD), categorical variables are reported as category counts and percentages. *P* values less than 0.05 were considered significant.

## Results

### Anatomic precision

**Fig. 2:**
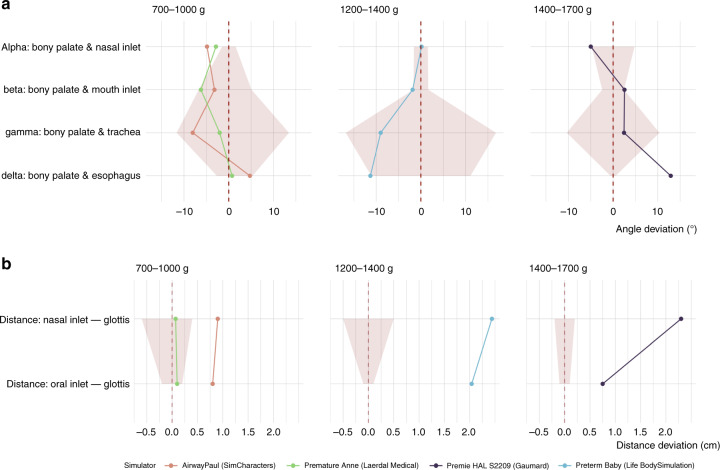
Airway dimensions of the tested simulators compared with reference values of human cadavers. **a** Airway angle deviation and **b** airway distance deviation in very low birth weight airway simulators compared to anatomical specimen of stillborn preterm infants in corresponding weight categories. The dotted red line shows the mean, the red ribbons the minimal and maximal airway angles and distances in the corresponding reference cadavers.

The spatial relationships between the oral cavity, the nasal inlet, larynx, and esophagus play a significant role during laryngoscopy as they define how the patient must be positioned, held, and what pressure and angle must be applied with the laryngoscope for successful airway maneuvers.^22^ We, therefore, measured airway angles in the four simulators and calculated the difference to the angles measured in reference cadavers according to weight categories. In addition, we measured the distance between the mouth entrance or nasal entrance and the glottis (Fig. 1) in the respective simulators and reference cadavers. Figure 2 shows that most angles were within the range of angles measured in the corresponding reference cadavers (red ribbon). In the simulators, Premature AirwayPaul (SIMCharacters) and Premature Anne (Laerdal Medical), the angle between the nasal inlet and the bony palate (alpha) was smaller than in the cadavers of according weight (−4.9° and −2.9° vs. −1.5° to 1.5°). In the simulator Premie HAL 2209 (Gaumard), the angle between the bony palate and the esophagus (delta) was much larger compared to the reference cadavers (12.9° vs. −0.6° to 0.6°) (Fig. 2a). The distances from the nasal inlet and mouth inlet to the glottis were within the range measured in the respective reference cadavers only in the Premature Anne simulator (Laerdal Medical). In the other three simulators, the distances were markedly too long (Fig. 2b).

### Simulator airway and lung physiology

#### Resistance and compliance

**Fig. 3:**
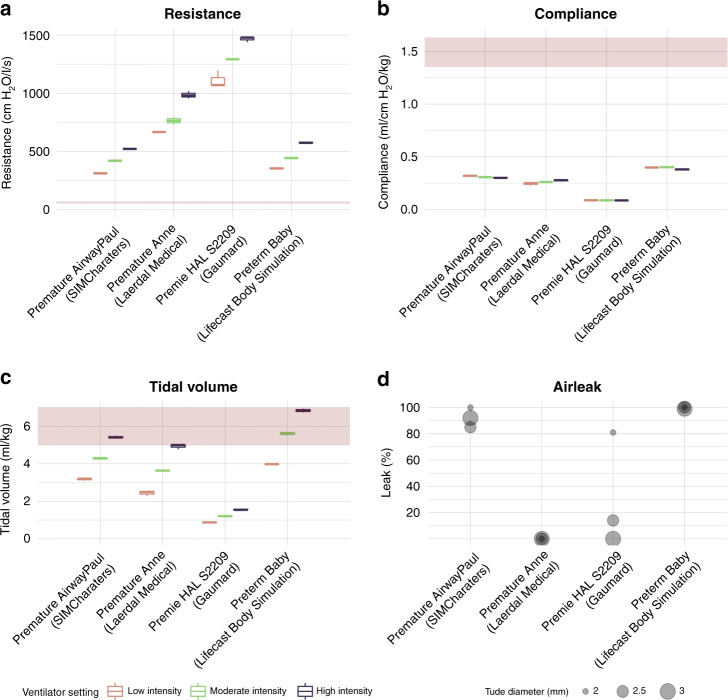
Airway and lung physiology of four very low birth weight airway simulators. The figures show the **a** resistance, **b** static compliance, and **c** tidal volume of the simulators at three ventilation intensities (low, moderate, high) together with the 95% confidence interval of reference values for very low birth weight infants (red area). The ventilator settings were chosen using a constant inspiratory time of 0.3 s, oxygen fraction of 0.21, frequency of 50/min, and a gas flow of 8 l/min. Pressure was chosen as follows: low intensity: peak inspiratory pressure (Pip) low (brown boxplots): 15 cm H_2_O, positive end-expiratory pressure (PEEP) 5 cm H_2_O, moderate intensity: Pip 20 cm H_2_O, PEEP 6 cm H_2_O (green boxplots), and high intensity: Pip 25 cm H_2_O, PEEP 7 cm H_2_O (purple boxplots). We performed each physiologic measurement three times. The bottom figure illustrates the **d** air leak for tubes of different diameters (2.0, 2.5, 3.0 mm) for each simulator at moderate intensity. Each parameter was measured three times.

Figure 3 shows the measured airway and lung parameters resistance, static compliance, and tidal volume per kg body weight for each simulator at three different intensities of mechanical ventilation together with the corresponding reference values.^23,24^ Compared to reference values for preterm infants, all simulator models had resistances that were up to 20 times higher (Premature Anne: 806 ± 143, Premature AirwayPaul: 417 ± 90 cm H_2_O/l/s, Premie HAL S2209: 1291 ± 157 cm H_2_O/l/s, Preterm Baby: 459 ± 96 cm H_2_O/l/s, Reference: 61 ± 26 cm H_2_O/l/s) (Fig. 3a), whereas the compliance was up to 19 times too low (Premature AirwayPaul: 0.308 ± 0.008 ml/cm H_2_O/kg, Premature Anne: 0.286 ± 0.015 ml/cm H_2_O/kg, Premie HAL S2209: 0.087 ± 0.001 ml/cm H_2_O/kg, Preterm baby: 0.393 ± 0.011 ml/cm H_2_O/kg, Reference: 1.49 ± 0.4 ml/cm H_2_O/kg) (Fig. 3b).^23,24^

#### Tidal volume

Only ventilation with the highest intensity using a high peak inspiratory pressure of 25 cm H_2_O generated tidal volumes similar to target tidal volumes during mechanical ventilation of preterm infants in three of the simulators (Premature Anne: 4.9 ± 0.1 ml/kg, Premature AirwayPaul: 6.2 ± 0.1 ml/kg, Preterm Baby: 7.1 ± 0.1 ml/kg, reference: 5–7 ml/kg).^25^ In the Premie HAL S2209 (Gaumard), very low tidal volumes were generated even with the most intensive ventilation setting Premie HAL S2209: 1.6 ± 0 ml/kg (Fig. 3c).

#### Air leak

Figure 3d illustrates the air leak for tubes of different diameters for each simulator. Simulators with low airway resistance had large air leaks. Premature Anne (Laerdal Medical) had no measurable air leak regardless of the tube diameter. In contrast, we measured leaks greater than 80% for both Premature AirwayPaul (SIMCharacters) and Preterm Baby (Lifecast Body Simulation) regardless of the tube diameter. For Premie HAL S2209 (Gaumard), the air leak was dependent on the tube diameter.

**Fig. 4:**
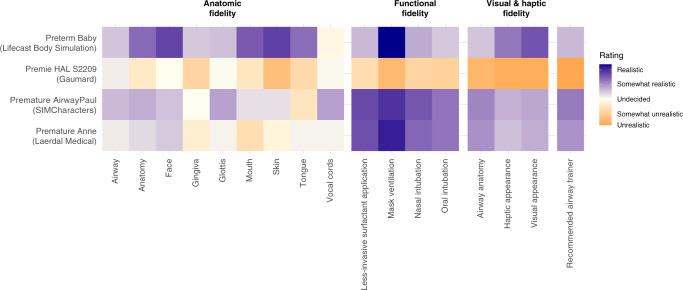
Subcategories of expert rating for each very low birth weight airway simulator. From left to right: anatomical fidelity, functional fidelity, visual and haptic fidelity, and recommendation level. Orange colors indicate poor rating values and poor recommendation levels.

### Expert rating

#### Experts

In total, 56 highly experienced experts from 11 centers (perinatal center level of care III and IV) participated in evaluating the simulators (STab. 1). Among the experts, 36 were neonatology fellows, 16 were consultants, and 3 were chief physicians. Participants had a mean ± SD professional experience of 18.0 ± 7.6 years. The Supplementary Table (STab. 1) shows details regarding the experts’ specific preterm infant airway skills.

#### Ratings

The experts rated the respective simulators with an overall mean ± SD rating score of Premature AirwayPaul (SIMCharacters) 5.1 ± 1.0, Premature Anne (Laerdal Medical) 4.9 ± 1.1, Premie HAL S2209 (Gaumard) 2.8 ± 1.0, and Preterm Baby (Lifecast Body Simulation) 5.0 ± 1.0. The total rating was calculated as the mean of the subcategories Anatomic Fidelity, Functional Fidelity, Visual and Haptic Appearance, and recommendation.

The heat map (Fig. 4) illustrates detailed information regarding the experts’ ratings for each subcategory of simulator fidelity and their recommendation to be used as a VLBW infant airway trainer. Premature Anne (Laerdal Medical) revealed weaknesses in mouth and skin, whereas Premature AirwayPaul (SIMCharacters) received a poor rating for the representation of the tongue. Preterm Baby (Lifecast) scored poorly for its vocal cords. Premie HAL (Gaumard) scored poor and medium ratings in almost all subcategories. Overall, on average, the simulator Premature AirwayPaul (SIMCharacters) received the best recommendation level (5.3 ± 1.4).

#### Experts’ and hospital characteristics as predictors of the total rating

**Fig. 5:**
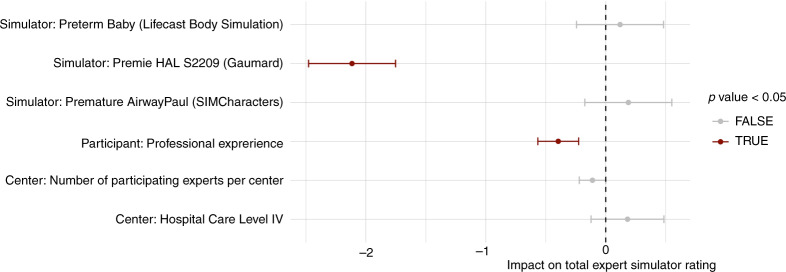
Linear multivariate regression model to analyze the impact of the experts’ and hospital characteristics and the simulator type on the total expert rating. The simulator type impact estimated by the model refers to Premature Anne (Laerdal, Stavanger, Norway), taken as the reference simulator.

Linear multivariate regression analysis showed that experts with more professional experience gave more unsatisfactory ratings (estimate = −0.4, *p* < 0.001) (Fig. 5) and confirmed the significantly lower rating of the Premie HAL S2209 (Gaumard) (estimate = −2.1, *p* < 0.001) compared to the other simulators. Hospital care level and the number of participants per center did not impact the total expert rating.

## Discussion

We investigated the anatomical precision, physiological characteristics of the airways and lungs, and the anatomic, functional, visual, and haptic fidelity of four VLBW airway simulators.

### Anatomic precision of the airway simulators

We conducted CT studies and compared angles and distances of critical airway structures in the sagittal plane with anatomical preparations of preterm infant cadavers of comparable gestational age and weight categories. This approach allowed the comparison of simulator anatomy with human references. We show that the airway angles of the simulators reflected fairly well the topographical relationships between crucial structures of the preterm infant airway. We found large individual ranges of the respective airway angles in the cadavers used as a reference, especially between the bony palate and the trachea (red ribbon in Fig. 2a). The distances from the nasal inlet and mouth inlet to the glottis were within the range measured in the respective reference cadavers only in the Premature Anne simulator (Laerdal Medical). In the other three simulators, the distances were markedly too long (Fig. 2b). However, we had to compare the Premature Anne Simulator (440 g) with heavier cadavers (700–1000 g), as no matching lighter cadavers were available. For this reason, we assume that the airway distances in Premature Anne (Laerdal Medical) may also be rather too long.

The simulators had too long distances, leading to unrealistically deep tube positioning in the simulators during oral or nasal intubation. Health-care providers may develop a misconception of how far a tube protrudes from a VLBW infant’s nose or mouth under real-life conditions (Fig. 2b). In summary, the simulators tested depicted the relative topographic relationships of important airway structures reasonably well, but the dimensions of the installed airways were too large in the simulators.

Schebesta et al. assessed high-fidelity adult patient simulators and two adult airway trainers’ similarity to human patients by measuring 14 predefined distances, two cross-sectional areas, and three upper airway volumes. They reported significant differences in pharyngeal airspace dimensions.^19^ A research group led by Cook and colleagues^9,26,27^ found evidence that devices performed differently depending on the type of simulator used. Blackburn et al.^18^ compared the anatomic accuracy of adult airway training manikins with humans and identified relevant inaccuracies in static dimensions. They concluded that this observation might imprecise airway device development, negatively affect training, and cause over-confidence in users. Critical voices question research on simulators, e.g., the development of new airway devices, since the validity is subject to substantial limitations due to the anatomical deviations from reality.^28^ However, due to the increasingly rare opportunities to learn complex airway maneuvers in VLBW infants, we believe simulators will play a crucial role in the future.

From the perspective of health-care providers, an optimal and contemporary VLBW simulator should be highly realistic, durable, and affordable at the same time. We still see the potential to improve the tested VLBW infant airway simulators by optimizing crucial airway structures’ angles and distances to facilitate highly realistic airway training to optimize effective skill transfer.

### Simulator physiology

The physiological characteristics of the simulators were highly different from the reference values of preterm infants in the corresponding weight categories (Fig. 3). Compliance was orders of magnitude too low in all simulators, while the resistance was much too high.^23^ Overall, the airways and lungs in the simulators are far too stiff and do not even remotely reflect the physiological conditions in VLBW preterm infants. Overly stiff simulator lungs, which only fill when ventilated with high pressures, could lead users to adopt excessively high pressures when ventilating VLBW infants. The Premie HAL S2209 (Gaumard) has only one lung on one side, resulting in asymmetric thorax excursions. The total compliance of the respiratory system is determined by both the lungs and chest wall. The lung compliance in extremely preterm infants varies, being very low soon after birth due to respiratory distress syndrome and changes during postnatal life and with interventions such as surfactant administrations. The compliance of the chest wall in extremely preterm infants is, however, very high. In comparison, the compliance of the chest wall of simulators is likely to be much lower, contributing to the total lower simulator compliance reported in this study.

The manufacturers do not claim that the installed lungs of the simulators have natural properties. However, natural resistance and compliance of the lungs during ventilation via a mask or endotracheal tube contribute to a realistic overall impression and should be emphasized in future preterm simulators.

### Expert rating

Experts evaluated the anatomic, functional, visual, and haptic fidelity of the four VLBW infant airway simulators by visual inspection, haptic testing, an inspection of airway pictures, and performing crucial airway maneuvers. The expert ratings showed that all simulators had individual strengths and weaknesses (Fig. 4). Three simulators received good overall ratings and scored similarly, only the Premie HAL S2209 (Gaumard) performed significantly worse. Sawyer et al. evaluated eight neonatal airway simulators, including two VLBW simulators for physical and functional fidelity, by a panel of neonatal healthcare professionals. They found significant differences between simulators^21^ and a high-fidelity rating of Premature Anne (Laerdal Medical). Analysis of our experts and hospital characteristics showed that experts with more professional experience gave more unsatisfactory ratings and were more critical of the simulators. This observation could be related to the personal experience in the care of VLBW preterm infants among experts with more professional experience, which could lead to a more critical evaluation of a simulator. In contrast, the level of care and the number of participants, as a correlate for the department’s size, had no significant impact on the assessment of the simulators.

### Limitations

We tested only a limited number of VLBW infant airway simulators. Other models with better characteristics may exist. Comparison with cadavers may be a potential bias, as dissection and postmortem tissue changes might cause deviations from the airway angles in living VLBW infants. Due to a lack of matching cadavers in Premature Anne’s weight class, the results regarding airway distances should be interpreted with caution. We placed all three tubes in all four simulators, accepting a significant discrepancy between simulator body dimension and tube diameter. Evaluation by experts is always subjective, but we obtained a wide range of opinions due to a large number of different centers and participants. We did not survey whether the experts had prior experience with any of the simulators, and therefore cannot completely rule out a potential bias.

## Conclusions

Three of the VLBW airway simulators tested had similar angles between crucial anatomical airway structures and anatomical specimens of preterm cadavers in corresponding weight categories. In contrast, the distances between the nasal inlet or the mouth aperture to the glottis were within a realistic range only in one simulator. The physiological characteristics of all simulator airways deviated significantly from preterm reference values concerning resistance and compliance. Only mechanical ventilation with very high peak inspiratory pressure generated tidal volumes in the simulators similar to reference values of ventilated preterm infants. Due to this severe deviation from natural conditions, we see a potential danger that users could become accustomed to a ventilation technique that is far too intensive. Three of the simulators performed well according to the expert rating, but all simulators showed individual weaknesses. In developing future VLBW infant simulators, manufacturers should take physiological aspects more into account and use the identified weaknesses to improve further the simulators’ anatomic accuracy, physiological properties, and physical and functional fidelity.

## Supplementary information

supplementary_material_legends

Supplementary stable 1

Supplementary sfigure 1
