# Supplementary material for: Anatomic accuracy, physiologic characteristics, and fidelity of very low birth weight infant airway simulators
Source: Pediatr Res. 2021 Nov 8;92(3):783–90. doi: 10.1038/s41390-021-01823-w (PMC8573578; doi:10.1038/s41390-021-01823-w)
Supplement: Supplementary file 1 — supplementary_material_legends [file 41390_2021_1823_MOESM1_ESM.docx]

**Supplementary Material**

**sFigure 1:** We showed experts in randomized order photos of the upper simulator airway, here exemplary for the glottis of A) Premature Anne (Laerdal Medical), B) Premature AirwayPaul (SIMCharacters), C) Premie HAL S2209 (Gaumard), and D) Preterm Baby (Lifecast Body Simulation). The photos were captured with a video laryngoscope Model C-MAC (Storz).
