# Supplementary material for: Anatomic accuracy, physiologic characteristics, and fidelity of very low birth weight infant airway simulators
Source: Pediatr Res. 2021 Nov 8;92(3):783–90. doi: 10.1038/s41390-021-01823-w (PMC8573578; doi:10.1038/s41390-021-01823-w)
Supplement: Supplementary file 2 — Supplementary stable 1 [file 41390_2021_1823_MOESM2_ESM.docx]

| **sTable 1: Experts and hospital characteristics** | | | | |
| --- | --- | --- | --- | --- |
| **Experts** |  | **n** | **(%)** |  |
| Professional status | Fellow | 36 | (66) |  |
|  | Consultant | 16 | (29) |  |
|  | Head Physician | 3 | (5) |  |
|  |  |  |  |  |
| Experience in positive pressure ventilation of VLBW infants | <20 | 1 | (2) |  |
|  | 20-50 | 0 | (0) |  |
|  | 51-100 | 5 | (9) |  |
|  | 101-200 | 10 | (18) |  |
|  | >200 | 39 | (71) |  |
|  |  |  |  |  |
| Number of intubations of VLBW infants | <20 | 4 | (7) |  |
|  | 20-50 | 10 | (18) |  |
|  | 50-100 | 15 | (27) |  |
|  | 100-200 | 10 | (18) |  |
|  | >200 | 16 | (29) |  |
|  |  |  |  |  |
| Number of less invasive surfactant applications in VLBW infants | <20 | 17 | (31) |  |
|  | 20-50 | 15 | (27) |  |
|  | 50-100 | 13 | (24) |  |
|  | 100-200 | 5 | (9) |  |
|  | >200 | 5 | (9) |  |
|  |  |  |  |  |
| **Hospital** |  |  |  |  |
| Perinatal care level | Level III | 32 | (58) |  |
|  | Level IV | 23 | (42) |  |
| VLBW: very low birth weight | | | | |
