# Supplementary figures and images for: Anatomic accuracy, physiologic characteristics, and fidelity of very low birth weight infant airway simulators
Source: Pediatr Res. 2021 Nov 8;92(3):783–90. doi: 10.1038/s41390-021-01823-w (PMC8573578; doi:10.1038/s41390-021-01823-w)

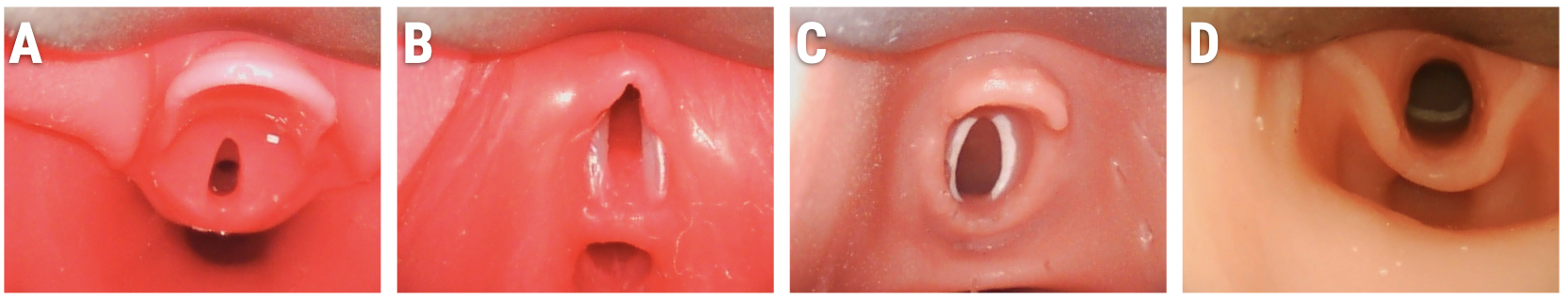

Supplement: Supplementary file 3 — Supplementary sfigure 1 [file 41390_2021_1823_MOESM3_ESM.jpeg]
